# Hemoglobin Changes After Long-Term Intermittent Work at High Altitude

**DOI:** 10.3389/fphys.2018.01552

**Published:** 2018-11-01

**Authors:** Almaz Akunov, Akylbek Sydykov, Turgun Toktash, Anara Doolotova, Akpay Sarybaev

**Affiliations:** ^1^Department of Mountain and Sleep Medicine and Pulmonary Hypertension, National Center of Cardiology and Internal Medicine, Bishkek, Kyrgyzstan; ^2^Kyrgyz Indian Mountain Biomedical Research Center, Bishkek, Kyrgyzstan; ^3^Excellence Cluster Cardio-Pulmonary System, Universities of Giessen and Marburg Lung Center, Member of the German Center for Lung Research (DZL), Justus Liebig University Giessen, Giessen, Germany; ^4^Medical Department, Kumtor Gold Company, Bishkek, Kyrgyzstan

**Keywords:** chronic intermittent hypoxia, high altitude, shift workers, hemoglobin, body mass index

## Abstract

Chronic high altitude hypoxia leads to an increase in red cell numbers and hemoglobin concentration. However, the effects of long-term intermittent hypoxia on hemoglobin concentration have not fully been studied. The aim of this study was to evaluate hemoglobin levels in workers commuting between an elevation of 3,800 m (2-week working shift) and lowland below 1,700 m (2 weeks of holiday). A total of 266 healthy males, aged from 20 to 69 years (mean age 45.9 ± 0.6 years), were included into this study. The duration of intermittent high altitude exposure ranged from 0 to 21 years. Any cardiac or pulmonary disorder was excluded during annual check-ups including clinical examination, clinical lab work (blood cell count, urine analysis, and biochemistry), ECG, echocardiography, and pulmonary function tests. The mean hemoglobin level in workers was 16.2 ± 0.11 g/dL. Univariate linear regression revealed an association of the hemoglobin levels with the years of exposure. Hemoglobin levels increased 0.068 g/dL [95% CI: 0.037 to 0.099, *p* < 0.001] for every year of intermittent high altitude exposure. Further, after adjusting for other confounding variables (age, living at low or moderate altitude, body mass index, and occupation) using multivariable regression analysis, the magnitude of hemoglobin level changes decreased, but remained statistically significant: 0.046 g/dL [95% CI: 0.005 to 0.086, *p* < 0.05]. Besides that, a weak linear relationship between hemoglobin levels and body mass index was revealed, which was independent of the years of exposure to high altitude (0.065 g/dL [95% CI: 0.006 to 0.124, *p* < 0.05]). We concluded that hemoglobin levels have a linear relationship with the exposure years spent in intermittent hypoxia and body mass index.

## Introduction

Last decades, the number of people traveling to high altitude is increasing in connection with economic or recreational purposes (West, 2012). High altitude environment poses many challenges, but exposure to alveolar hypoxia is the most prominent among them (Burtscher et al., 2018). The ambient hypoxia triggers a number of physiologic responses including hyperventilation, increased resting heart rate and stimulation of erythrocyte production with the goal of maintaining the oxygen content of arterial blood at or above sea level values (West, 2004). In permanent high altitude residents, exposure to chronic hypoxia leads to an increase in erythrocyte numbers and hemoglobin concentration (Leon-Velarde et al., 2000).

A large group of people, such as workers of mining companies, employees of road construction companies or military divisions in high-altitude border areas are exposed repetitively to high altitude over a long time by commuting between high altitude and lowland (Powell and Garcia, 2000; Richalet et al., 2002; West, 2002; Farias et al., 2013). However, the effects of the long-term exposure to intermittent hypoxia on hemoglobin concentration have been less well studied. Only few studies have assessed hematological changes induced by long-term intermittent high altitude exposure. Some studies reported a marked increase in hemoglobin (or hematocrit) levels in response to long-term exposure to intermittent high altitude (Gunga et al., 1996; Heinicke et al., 2003), whereas others did not find a significant effect (Richalet et al., 2002; Brito et al., 2007, 2018). Therefore, the aim of the current study was to determine the association of long-term intermittent high altitude exposure with hemoglobin levels as well as to explore the nature of this possible relationship.

## Materials and Methods

### Subjects and Study Design

This is a cross-sectional study of mine employees exposed repetitively to high altitude for long periods of time. All of them work at the gold mine run by the Kumtor Gold Company (Centerra Gold Inc., Canada). Mine employees are transported to the mine site by bus, and the ascent lasts 7 h in total.

Every worker undergoes an annual medical checkup in a specially designated clinic in Bishkek (Kyrgyzstan, 760 m), which includes physical examination, clinical lab work (complete blood count, urine analysis, and biochemistry), ECG, echocardiography, exercise testing and spirometry. All the male employees of the Kumtor Gold Company, who had medical checkup during October–December 2016, were enrolled into the study. The inclusion criteria were male sex, uninterrupted working at high altitude in 14×14 shifts (14 days of working at altitude followed by a resting period of 14 days at lowland) and a healthy status without serious cardiopulmonary comorbidities. The exclusion criteria were chronic obstructive pulmonary disease, moderate or severe hypertension, and coronary heart disease.

In total, 266 healthy males (aged 45.9 ± 0.6 years) were included into the study. Subjects were truck drivers, gold mill operators, camp and kitchen staff, security personnel, and engineers. All subjects commuted between high altitude and living place (760 m or 1,600 m) in a 14×14 shift regimen. The miners slept at 3,800 m and worked a 12-h day shift at 3,800 to 4,000 m, only few subjects (operators of drilling rigs) went to 4,500 m.

Written informed consent was obtained from all participants in accordance with the Declaration of Helsinki. This study was approved by the Research Ethics Committee of the National Center of Cardiology and Internal Medicine, Bishkek, Kyrgyzstan.

### Measured Variables

Measures were taken once at low altitude in a specially designated clinic in Bishkek during the annual medical check-up. Usually, the annual medical check-up is carried out 1 week after descent. Any cardiac or pulmonary disorder was excluded based on the results of clinical examination, clinical lab work (blood cell count, urine analysis, and biochemistry), ECG, echocardiography, and pulmonary function tests.

#### General Data

Weight and height measured by means of a digital weight scale and a stadiometer with each participant barefoot and wearing underwear. Body mass index values were calculated as weight in kilograms divided by height in meters squared and rounded to 1 decimal place.

#### Hematological Measurements

Complete blood count was performed by an automated hematology analyser Mindray BC-2300 (Guangzhou Shihai Medical Equipment Co., Ltd., China) using 15 μm of EDTA whole venous blood according to the manufacturer’s instructions. Hematocrit, concentration of hemoglobin, erythrocytes, leukocytes, and platelets were measured. The analyser uses electrical impedance method for cell counting and cyanide free method for hemoglobin detection. Red blood cell indices included hemoglobin concentration and mean corpuscular volume.

### Data Analysis

Statistical analysis was performed using SPSS version 23.0 for Windows (IBM, Chicago, IL, United States). Data are expressed as mean ± standard error. The Kolmogorov–Smirnov test was used to assess the normality of distribution of quantitative variables. All quantitative variables were distributed normally. Linearity was assessed through examination of various bivariate scatterplots. Linear regression (univariate and multivariable) analysis was used to check the association between hemoglobin levels and other parameters. Differences between different BMI categories were assessed by one-way ANOVA followed by Tukey’s multiple comparisons *post hoc* test. For comparing proportions, we used chi-squared test. For linear trend in quantitative variables we used one-way ANOVA linear contrast method. The values *P* < 0.05 were considered as statistically significant. Statistical power analysis was performed using G^∗^Power 3.1.

## Results

**FIGURE 1:**
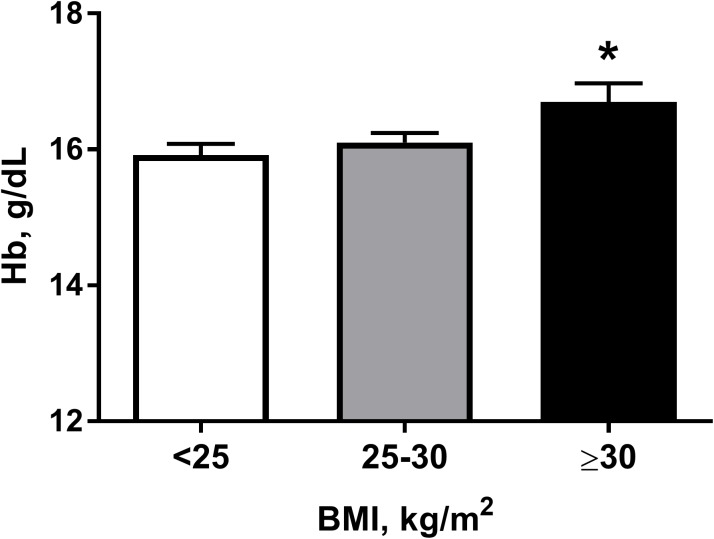
Hemoglobin (Hb) levels in subjects according to their body mass index (BMI) category: normal weight (<25), overweight (25–30), and obese (≥30). ^∗^*P* < 0.05 for differences between normal weight and obese subjects. Data presented as mean ± SEM. Statistical analysis was carried out using one-way ANOVA followed by Tukey’s multiple comparison test.

Characteristics describing the subjects are provided in Table 1. One hundred and eighty-four workers were residents of moderate altitude (1,600 m); the remaining 82 men were residents of low altitude (760 m). The duration of exposure to intermittent high altitude ranged from 0 to 21 years. The average record of service was 10.1 years. The majority of subjects were ethnic Kyrgyz (92%), but there were some Caucasians too (8%). Prevalence of obesity in the sample was 16.1 ± 2.4%. The mean hemoglobin level in workers was 16.2 ± 0.11 g/dL (Table 1). Table 2 shows higher hemoglobin levels, as well as age and BMI, in subjects with longer duration of high altitude exposure. It is clear that hemoglobin levels are higher when the duration of exposure is longer (*p* < 0.001). Notably, the differences in age and BMI were also statistically significant; therefore, we cannot ascribe the differences in hemoglobin levels to the effects of high altitude alone. Indeed, hemoglobin values were significantly higher in obese subjects compared to those with normal weight (Figure 1).

**Table 1:** Anthropometric hematological characteristics of the subjects (*n* = 266).

Variables	Mean ± SEM
Age, years	45.9 ± 0.6
Body mass index, kg/m^2^	26.8 ± 0.22
Obesity, %	16.1 ± 2.4
Kyrgyz ethnicity, %	91.4 ± 1.7
Job (outdoor vs. indoor)	73.1 ± 2.7
Duration of intermittent high altitude exposure, years	10.1 ± 0.4
Erythrocytes, 10^12^/L	5.12 ± 0.03
Hemoglobin, g/dL	16.2 ± 0.11
Hematocrit, %	48 ± 0.3
Mean corpuscular volume, fL	93.9 ± 0.3
Leukocytes, 10^9^/L	7.28 ± 0.12
Platelets, 10^9^/L	245 ± 3.5


**Table 2:** Distribution of the hemoglobin levels, age, body mass index, and residence place of the subjects according to the years of high altitude exposure.

Variables	Intermittent high altitude exposure, years					*P*
	0	1–5	6–10	11–15	>15	
Hb, g/dL	14.9 ± 0.25	15.9 ± 0.27	16.1 ± 0.18	16.5 ± 0.26	16.6 ± 0.2	<0.001
Age, years	37.3 ± 1.6	39.2 ± 1.8	44.4 ± 0.9	50.1 ± 1.3)	53.3 ± 0.8	<0.001
BMI, kg/m^2^	24.3 ± 0.9	26.4 ± 0.6	26.7 ± 0.4	27.6 ± 0.5	27.7 ± 0.4	<0.01
Residents of moderate altitude, %	88 ± 8	53 ± 9	68 ± 5	66 ± 8	76 ± 6	NS


*Data are presented as mean ± SEM (n = 266). Hb, hemoglobin; BMI, body mass index. p for linearity (hemoglobin, age, BMI) was determined using one-way ANOVA linear contrast analysis; p for trend (residents) was determined using chi^2^-criterion.*

**FIGURE 2:**
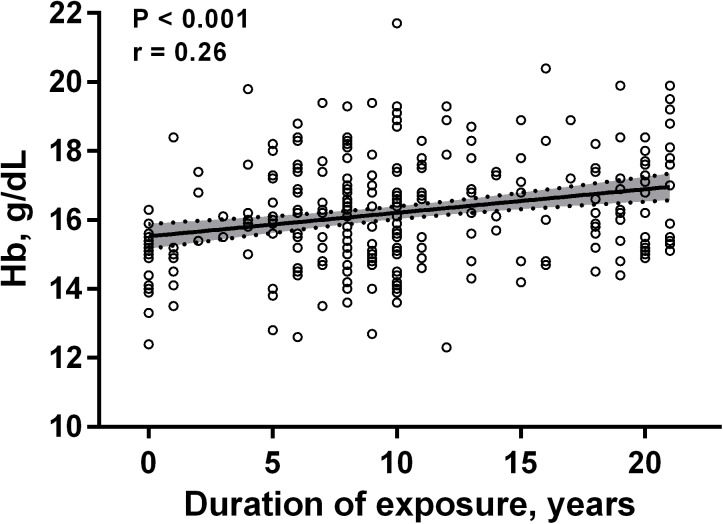
Scatter plot of correlation between the hemoglobin (Hb) levels and the years of exposure to intermittent high altitude. The best-fit line is shown, and the shaded area represents the 95% confidence intervals (*n* = 266).

Univariate linear regression revealed an association of the hemoglobin levels with the years of exposure (Figure 2). In a univariate regression model, every consecutive year was associated with an increase in hemoglobin of 0.068 g/dL [95% CI: 0.037 to 0.099, *p* < 0.001].

Further, after adjusting for other variables (age, living at low or moderate altitude, BMI, and occupation) using multivariable regression analysis, the magnitude of hemoglobin level changes decreased, but remained statistically significant: 0.046 g/dL [95% CI: 0.005 to 0.086, *p* < 0.05]. Despite a significant association of the hemoglobin levels with age by univariate regression analysis, it failed to prove the significance after adjusting for the rest variables (BMI, years of exposure, altitude of residence (0.006 g/dL [95% CI -0.024 to -0.035]) (Table 3). However, BMI and duration of exposure retained to have a weak but significant relationship with hemoglobin levels (0.065 g/dL [95% CI: 0.006 to 0.124] and 0.046 g/dL [95% CI: 0.005 to 0.086], respectively) (Table 3).

**Table 3:** Associations between hemoglobin levels and other variables by univariate and multivariable analysis.

Variables	Univariate			Multivariable*		
	*b*	95% CI	*p*	*b*	95% CI	*p*
Age, years	0.027	0.006–0.048	<0.05	0.006	-0.024 – 0.035	NS
BMI, kg/m^2^	0.084	0.027–0.141	<0.01	0.065	0.006 – 0.124	<0.05
Years of intermittent high altitude exposure	0.068	0.037–0.099	<0.001	0.046	0.005 – 0.086	<0.05
Residence at moderate altitude	0.023	-0.39 – 0.44	NS	-0.061	-0.239 – 0.117	NS


*BMI, body mass index. ^∗^adjusted for the other variables. R^2^= 0.07.*

## Discussion

To our best knowledge, this is the first study performed on a large population of Kyrgyz workers intermittently exposed to high altitude for a very long period up to 21 years. We showed that there is a statistically significant correlation between the hemoglobin levels and the years of exposure. Using multivariable regression analysis, we showed that hemoglobin levels increase by an average of 0.046 g/dL for every consecutive year of intermittent high altitude exposure, after adjusting for other variables (age, living at low or moderate altitude, BMI, and occupation). Interestingly, the residence at low or moderate altitudes did not affect the hemoglobin levels. This may be due to the relatively small difference in the altitude of residence (less than 900 m).

Chronic high altitude hypoxia leads to an increase in red cell numbers and hemoglobin concentration. Previous studies have shown that permanent high altitude residents possess elevated hemoglobin levels and hematocrit values (Leon-Velarde et al., 2000). In sea-level residents, hematocrit and hemoglobin concentration were elevated after exposure to 3,550-m altitude for 8 months; however, none of the parameters reached pathological values (Siques et al., 2007). Only few studies have assessed hematological changes induced by long-term intermittent high altitude exposure in a comprehensive manner. An earlier study in Chilean workers exposed to intermittent high altitude for >5 years revealed hemoglobin values that were comparable to those reported in the literature for high altitude populations (Gunga et al., 1996). Similarly, increased hemoglobin concentration and hematocrit values (15.8 ± 1.2 g/dL and 46.2 ± 3.8%, respectively) were shown in Chilean army officers exposed to intermittent hypoxia for about 22 years after 3 days following descent to sea level (Heinicke et al., 2003). Remarkably, hemoglobin concentration and hematocrit values (16.5 ± 0.9 g/dL and 48.1 ± 2.9%, respectively) measured at high altitude were comparable to those found in permanent high altitude residents (Heinicke et al., 2003). Another study conducted in a group of Chilean army officers exposed to intermittent high altitude for at least 12 years (50% had been >25 years at altitude) reported a smaller increase in hemoglobin concentration and hematocrit values (15.1 ± 1.0 g/dL and 45.02 ± 2.7%, respectively) measured on day 1 following descent to sea level (Brito et al., 2007). Similarly, a period of 2.5-year exposure to intermittent hypoxia induced a significant hematocrit increase in Chilean miners, which was, however, lower than what is observed in permanent high altitude residents (Richalet et al., 2002). A recent cross-sectional study in healthy Chilean male miners working at an altitude of 4,400 or 4,800 m for, on average, 14 years reported mean hematocrit and hemoglobin values of 47.6 ± 0.3% and 16.2 ± 0.1 g/dL, respectively, with none of the subjects having pathological values (Brito et al., 2018). In our study, hemoglobin concentration and hematocrit values in shift workers were higher than those observed in sea level residents, but were lower than values reported in Aymara native residents at 3,800–4,065 m (Beall et al., 1998).

Various factors may be responsible for the discrepancy. A meta-analysis and a Monte Carlo simulation on the extracted data showed that red cell volume expansion for a given duration of exposure is dependent on the altitude (Rasmussen et al., 2013). The authors suggested that, at altitudes above 4,000 m, exposure time must exceed 2 weeks to exert a significant effect and that the magnitude of the erythropoietic response depends on the initial red cell volume (Rasmussen et al., 2013). In addition, hemoglobin mass returns to baseline sea level values in 2–3 weeks following descent to sea level (Wachsmuth et al., 2013). Moreover, the mining companies and military divisions in different countries implement various commuting patterns complicating the interpretation of the hematological changes induced by the long-term intermittent high altitude exposure. The most common shift patterns range from 4×3 days to 28×28 days (Gunga et al., 1996; Richalet et al., 2002; Heinicke et al., 2003; Sarybaev et al., 2003; Brito et al., 2007; Vinnikov et al., 2011). Furthermore, ethnic differences have been shown in hematological responses in high altitude residents (Beall, 2006). If ethnicity affects hematological responses to chronic intermittent high altitude exposure is not known.

Another explanation could be the various duration of stay at high altitude and the elevation achieved in different studies Therefore, a term “hypoxic dose” has recently been introduced as a new metric incorporating both altitude and total exposure time to measure an increase in hemoglobin mass because of intermittent hypoxic training or intermittent hypoxic exposure (Garvican-Lewis et al., 2016a). The models suggest that hypoxic exposure in excess of 250 km h is sufficient to produce an increase in hemoglobin mass (Garvican-Lewis et al., 2016b). In our study, the workers were exposed to the hypoxic dose of about 1,300 km h, which is considered to be sufficient to induce an increase in hemoglobin levels.

Univariate analysis showed a relationship of hemoglobin levels with aging; however, after adjusting for other variables, the value ceased to be significant. These results are in line with the findings from other studies. Thus, no significant hematocrit change over 11.6 years was demonstrated in a recent prospective study (Carallo et al., 2011). In another study, while fibrinogen, another blood viscosity parameter, increased with age, the hemoglobin level on the contrary slightly decreased in the elderly subjects (Coppola et al., 2000). Notably, we revealed a significant relation of hemoglobin levels with the BMI in workers exposed to intermittent high altitude for a long period. It is in accordance with other studies that demonstrated a positive association between hemoglobin and BMI in lowland populations (Micozzi et al., 1989; Skjelbakken et al., 2010). In contrast, no correlation between hemoglobin levels and BMI was reported by others (Ghadiri-Anari et al., 2014). Nevertheless, in a recent prospective study of high-altitude mine workers in Peru, male gender, duration of the intermittent high altitude exposure and BMI were independent predictors of hemoglobin level changes (Mejia et al., 2017). The underlying mechanisms remain poorly understood. However, a negative effect of BMI on oxygen saturation was demonstrated in Chinese Han young males during high altitude acclimatization (Peng et al., 2013). Interestingly, a negative association between BMI and blood oxygenation was also found in healthy high altitude residents in Peru (Pereira-Victorio et al., 2014; Miele et al., 2016). These data suggest that shift workers with higher BMI will have a greater increase in hemoglobin during chronic intermittent high altitude exposure.

It has been shown that borderline polycythemia (hematocrit above 50%) was associated with increased mortality from coronary heart disease (Kunnas et al., 2009). However, the connection between hemoglobin and cardiovascular diseases is complex and is still not clear (Brown et al., 2001; Frackiewicz et al., 2018). We have to admit that prolonged intermittent high altitude hypoxia can provoke cardiovascular events in patients with clinical or subclinical coronary atherosclerosis because of blood rheology changes. Interestingly, accumulating evidence demonstrates that short-term daily sessions of hypoxia alternating with equal durations of normoxia for 2–3 weeks exert beneficial effects on various cardiovascular diseases (Serebrovskaya and Xi, 2016), thus potentially opposing deleterious effects of polycythemia. At the same time, the increase in hemoglobin levels due to chronic intermittent high altitude exposure should not lead to increased probability of cardiovascular events *per se*.

Although the literature on the relation between changes in hemoglobin concentration and cardiovascular disorders in subjects exposed to long-term intermittent high altitude hypoxia is very scarce, we believe that such a small, though statistically significant, increase in hemoglobin concentration represents an adaptive response rather than a risk factor for cardiovascular diseases. An annual increase in hemoglobin level by 0.046 g/dL means that for every 10 years of work at intermittent high altitude hypoxia hemoglobin levels will rise, on average, less than 0.5 g/dL. Indeed, in most of the subjects chronically exposed to intermittent high altitude, hemoglobin concentration and hematocrit reach intermediate values that are higher than those in sea level residents but lower than those in healthy high altitude dwellers (Richalet et al., 2002; Brito et al., 2007). It is unlikely that this rather small increase in hemoglobin levels would have any significant pathological effects (Burtscher, 2014; Corante et al., 2018).

Additionally, another issue related to hemoglobin levels at high-altitude is the change in skeletal muscle mass, since muscles are the main consumer of oxygen in the body. While some studies indicated some decrease in skeletal muscle mass at high-altitude exposure (Hoppeler et al., 1990; MacDougall et al., 1991; Mizuno et al., 2008), the others were not able to show significant loss of muscle mass (Lundby et al., 2004; D’Hulst et al., 2016; Jacobs et al., 2016). Differences in the hypoxic doses may account for this discrepancy. Thus, it has been suggested that a minimum hypoxic exposure of 5,000 km h is required for hypoxia-induced muscle atrophy to develop (D’Hulst and Deldicque, 2017). Although, we have not measured the skeletal muscle fiber cross sectional area changes in the workers, it seems unlikely that the relatively low hypoxic dose in our study can significantly affect the skeletal muscle.

Another kind of intermittent hypoxia which deserves further commentary is obstructive sleep apnea syndrome (OSAS). OSAS is a pathological condition characterized by recurrent or cyclic short periods of isobaric hypoxia and asphyxia during sleep (coupled with oxygen desaturation), often more than 60 times per hour (Neubauer, 2001). Such frequent fluctuations of oxygen saturation lead to sympathetic overactivity, increased oxidative stress, and activation of inflammatory response pathways (Mansukhani et al., 2014; Passali et al., 2015; de Lima et al., 2016). In addition to hypoxemia, these events are associated with significant hypercapnia. Consequently, OSAS is an independent and well-known major risk factor for various cardiovascular diseases, including hypertension, stroke, myocardial infarction, and congestive heart failure (Kendzerska et al., 2014; Drager et al., 2015; Bauters et al., 2016). In contrast, chronic intermittent hypoxic exposure in miners involves prolonged cycles of hypobaric hypoxia alternating with normobaric normoxia. The frequency of high altitude intermittent hypoxia is usually one to four times a month, which does not have such unfavorable effects on the body. Thus, as previously pointed out (Richalet et al., 2002), OSAS is quite different from the chronic intermittent hypoxic exposure in miners.

One of the limitations of this study is its cross-sectional nature. Further, most of the subjects were permanent residents of a moderate altitude, so the difference in altitude between the residence place and working altitude was relatively small. Nevertheless, the strengths of our study include high number of subjects, which provided the high statistical power, and larger duration of intermittent high altitude exposure than in most other studies.

## Conclusion

In summary, this study adds to the growing body of knowledge concerning the physiology of long-term intermittent high altitude exposure. We defined for the first time hemoglobin levels in Kyrgyz shift workers commuting between high altitude and lowland. Further, our study provides evidence that hemoglobin levels have a linear relationship with years of intermittent high altitude exposure and BMI. Apparently, in chronic intermittent hypoxia exposure even over longer periods, excessive erythrocytosis does not represent a major problem for healthy shift workers. Our findings might have important implications for occupational medical surveillance to monitor health status of the workers exposed to chronic intermittent high altitude. We hope that our study would encourage further research to explore the long-term consequences of this unique biological condition.

## Author Contributions

AA, AbS, and ApS conceived and designed the study, drafted the manuscript, and provided overall supervision. AA, AbS, TT, and AD performed the data acquisition, analysis, or interpretation. AA, AbS, TT, AD, and ApS critically revised important intellectual content in the manuscript and approved the final version of the manuscript.

## Conflict of Interest Statement

TT and AD were employed by the Kumtor Gold Company. The remaining authors declare that the research was conducted in the absence of any commercial or financial relationships that could be construed as a potential conflict of interest.

## References

[B1] Bauters F. Rietzschel E. R. Hertegonne K. B. Chirinos J. A. (2016). The link between obstructive sleep apnea and cardiovascular disease. *Curr. Atheroscler. Rep.* 18:1. 10.1007/s11883-015-0556-z 26710793

[B2] Beall C. M. (2006). Andean, Tibetan, and Ethiopian patterns of adaptation to high-altitude hypoxia. *Integr. Comp. Biol.* 46 18–24. 10.1093/icb/icj004 21672719

[B3] Beall C. M. Brittenham G. M. Strohl K. P. Blangero J. Williams-Blangero S. Goldstein M. C. (1998). Hemoglobin concentration of high-altitude Tibetans and Bolivian Aymara. *Am. J. Phys. Anthropol.* 106 385–400. 10.1002/(SICI)1096-8644(199807)106:3<385::AID-AJPA10>3.0.CO;2-X 9696153

[B4] Brito J. Siques P. Leon-Velarde F. De La Cruz J. J. Lopez V. Herruzo R. (2007). Chronic intermittent hypoxia at high altitude exposure for over 12 years: assessment of hematological, cardiovascular, and renal effects. *High Alt. Med. Biol.* 8 236–244. 10.1089/ham.2007.8310 17824824

[B5] Brito J. Siques P. López R. Romero R. León-Velarde F. Flores K. (2018). Long-Term intermittent work at high altitude: right heart functional and morphological status and associated cardiometabolic factors. *Front. Physiol.* 9:248. 10.3389/fphys.2018.00248 29623044 PMC5874329

[B6] Brown D. W. Giles W. H. Croft J. B. (2001). Hematocrit and the risk of coronary heart disease mortality. *Am. Heart J.* 142 657–663. 10.1067/mhj.2001.118467 11579356

[B7] Burtscher M. (2014). Effects of living at higher altitudes on mortality: a narrative review. *Aging Dis.* 5 274–280. 10.14336/ad.2014.0500274 25110611 PMC4113517

[B8] Burtscher M. Gatterer H. Burtscher J. Mairbaurl H. (2018). Extreme terrestrial environments: life in thermal stress and hypoxia. a narrative review. *Front. Physiol.* 9:572. 10.3389/fphys.2018.00572 29867589 PMC5964295

[B9] Carallo C. Irace C. De Franceschi M. S. Coppoletta F. Tiriolo R. Scicchitano C. (2011). The effect of aging on blood and plasma viscosity. An 11.6 years follow-up study. *Clin. Hemorheol. Microcirc.* 47 67–74. 10.3233/ch-2010-1367 21321410

[B10] Coppola L. Caserta F. De Lucia D. Guastafierro S. Grassia A. Coppola A. (2000). Blood viscosity and aging. *Arch. Gerontol. Geriatr.* 31 35–42. 10.1016/S0167-4943(00)00063-7 10989162

[B11] Corante N. Anza-Ramirez C. Figueroa-Mujica R. Macarlupu J. L. Vizcardo-Galindo G. Bilo G. (2018). Excessive erythrocytosis and cardiovascular risk in andean highlanders. *High Alt. Med. Biol.* 19 221–231. 10.1089/ham.2017.0123 29782186 PMC6157350

[B12] de Lima F. F. Mazzotti D. R. Tufik S. Bittencourt L. (2016). The role inflammatory response genes in obstructive sleep apnea syndrome: a review. *Sleep Breath* 20 331–338. 10.1007/s11325-015-1226-7 26201496

[B13] D’Hulst G. Deldicque L. (2017). Human skeletal muscle wasting in hypoxia: a matter of hypoxic dose? *J. Appl. Physiol.* 122 406–408. 10.1152/japplphysiol.00264.2016 27742801

[B14] D’Hulst G. Ferri A. Naslain D. Bertrand L. Horman S. Francaux M. (2016). Fifteen days of 3,200 m simulated hypoxia marginally regulates markers for protein synthesis and degradation in human skeletal muscle. *Hypoxia* 4 1–14. 10.2147/hp.s101133 27800505 PMC5085286

[B15] Drager L. F. Polotsky V. Y. O’Donnell C. P. Cravo S. L. Lorenzi-Filho G. Machado B. H. (2015). Translational approaches to understanding metabolic dysfunction and cardiovascular consequences of obstructive sleep apnea. *Am. J. Physiol. Heart Circ. Physiol.* 309 H1101–H1111. 10.1152/ajpheart.00094.2015 26232233 PMC4816265

[B16] Farias J. G. Jimenez D. Osorio J. Zepeda A. B. Figueroa C. A. Pulgar V. M. (2013). Acclimatization to chronic intermittent hypoxia in mine workers: a challenge to mountain medicine in Chile. *Biol. Res.* 46 59–67. 10.4067/s0716-97602013000100009 23760416

[B17] Frackiewicz J. Wlodarek D. Brzozowska A. Wierzbicka E. Slowinska M. A. Wadolowska L. (2018). Hematological parameters and all-cause mortality: a prospective study of older people. *Aging Clin. Exp. Res.* 30 517–526. 10.1007/s40520-017-0791-y 28664457 PMC5911276

[B18] Garvican-Lewis L. A. Sharpe K. Gore C. J. (2016a). Last word on viewpoint: time for a new metric for hypoxic dose? *J. Appl. Physiol.* 121:359. 10.1152/japplphysiol.00457.2016 27451277

[B19] Garvican-Lewis L. A. Sharpe K. Gore C. J. (2016b). Time for a new metric for hypoxic dose? *J. Appl. Physiol.* 121 352–355. 10.1152/japplphysiol.00579.2015 26917695

[B20] Ghadiri-Anari A. Nazemian N. Vahedian-Ardakani H.-A. (2014). Association of body mass index with hemoglobin concentration and iron parameters in Iranian population. *ISRN Hematol.* 2014:525312. 10.1155/2014/525312 24665367 PMC3934448

[B21] Gunga H. C. Rocker L. Behn C. Hildebrandt W. Koralewski E. Rich I. (1996). Shift working in the Chilean Andes ( > 3,600 m) and its influence on erythropoietin and the low-pressure system. *J. Appl. Physiol.* 81 846–852. 10.1152/jappl.1996.81.2.846 8872655

[B22] Heinicke K. Prommer N. Cajigal J. Viola T. Behn C. Schmidt W. (2003). Long-term exposure to intermittent hypoxia results in increased hemoglobin mass, reduced plasma volume, and elevated erythropoietin plasma levels in man. *Eur. J. Appl. Physiol.* 88 535–543. 10.1007/s00421-002-0732-z 12560952

[B23] Hoppeler H. Kleinert E. Schlegel C. Claassen H. Howald H. Kayar S. R. (1990). Morphological adaptations of human skeletal muscle to chronic hypoxia. *Int. J. Sports Med.* 11(Suppl. 1) S3–S9. 10.1055/s-2007-1024846 2323861

[B24] Jacobs R. A. Lundby A. K. Fenk S. Gehrig S. Siebenmann C. Fluck D. (2016). Twenty-eight days of exposure to 3454 m increases mitochondrial volume density in human skeletal muscle. *J. Physiol.* 594 1151–1166. 10.1113/jp271118 26339730 PMC4771777

[B25] Kendzerska T. Gershon A. S. Hawker G. Leung R. S. Tomlinson G. (2014). Obstructive sleep apnea and risk of cardiovascular events and all-cause mortality: a decade-long historical cohort study. *PLoS Med.* 11:e1001599. 10.1371/journal.pmed.1001599 24503600 PMC3913558

[B26] Kunnas T. Solakivi T. Huuskonen K. Kalela A. Renko J. Nikkari S. T. (2009). Hematocrit and the risk of coronary heart disease mortality in the TAMRISK study, a 28-year follow-up. *Prev. Med.* 49 45–47. 10.1016/j.ypmed.2009.04.015 19409924

[B27] Leon-Velarde F. Gamboa A. Chuquiza J. A. Esteba W. A. Rivera-Chira M. Monge C. C. (2000). Hematological parameters in high altitude residents living at 4,355, 4,660, and 5,500 meters above sea level. *High Alt. Med. Biol.* 1 97–104. 10.1089/15270290050074233 11256567

[B28] Lundby C. Pilegaard H. Andersen J. L. van Hall G. Sander M. Calbet J. A. (2004). Acclimatization to 4100 m does not change capillary density or mRNA expression of potential angiogenesis regulatory factors in human skeletal muscle. *J. Exp. Biol.* 207(Pt 22) 3865–3871. 10.1242/jeb.01225 15472017

[B29] MacDougall J. D. Green H. J. Sutton J. R. Coates G. Cymerman A. Young P. (1991). Operation Everest II: structural adaptations in skeletal muscle in response to extreme simulated altitude. *Acta Physiol. Scand.* 142 421–427. 10.1111/j.1748-1716.1991.tb09176.x 1927554

[B30] Mansukhani M. P. Kara T. Caples S. M. Somers V. K. (2014). Chemoreflexes, sleep apnea, and sympathetic dysregulation. *Curr. Hypertens. Rep.* 16:476. 10.1007/s11906-014-0476-2 25097113 PMC4249628

[B31] Mejia C. R. Quinones-Laveriano D. M. Gomero R. Perez-Perez L. (2017). [Hemoglobin changes (Hb) in miners exposed to high altitude and associated factors]. *Gac. Med. Mex.* 153 166–172. 28474702

[B32] Micozzi M. S. Albanes D. Stevens R. G. (1989). Relation of body size and composition to clinical biochemical and hematologic indices in US men and women. *Am. J. Clin. Nutr.* 50 1276–1281. 10.1093/ajcn/50.6.1276 2596419

[B33] Miele C. H. Schwartz A. R. Gilman R. H. Pham L. Wise R. A. Davila-Roman V. G. (2016). Increased cardiometabolic risk and worsening hypoxemia at high altitude. *High Alt. Med. Biol.* 17 93–100. 10.1089/ham.2015.0084 27281472 PMC4913510

[B34] Mizuno M. Savard G. K. Areskog N. H. Lundby C. Saltin B. (2008). Skeletal muscle adaptations to prolonged exposure to extreme altitude: a role of physical activity? *High Alt. Med. Biol.* 9 311–317. 10.1089/ham.2008.1009 19115916

[B35] Neubauer J. A. (2001). Invited review: Physiological and pathophysiological responses to intermittent hypoxia. *J. Appl. Physiol.* 90 1593–1599. 10.1152/jappl.2001.90.4.1593 11247965

[B36] Passali D. Corallo G. Yaremchuk S. Longini M. Proietti F. Passali G. C. (2015). Oxidative stress in patients with obstructive sleep apnoea syndrome. *Acta Otorhinolaryngol. Ital.* 35 420–425. 10.14639/0392-100x-895 26900248 PMC4755047

[B37] Peng Q. Q. Basang Z. Cui C. Y. Li L. Qian J. Gesang Q. (2013). Physiological responses and evaluation of effects of BMI, smoking and drinking in high altitude acclimatization: a cohort study in Chinese Han young males. *PLoS One* 8:e79346. 10.1371/journal.pone.0079346 24260204 PMC3832642

[B38] Pereira-Victorio C. J. Huamanquispe-Quintana J. Castelo-Tamayo L. E. (2014). [Arterial blood gases in clinically healthy adults living at 3,350 meters of altitude]. *Rev. Peru. Med. Exp. Salud. Pub.* 31 473–479. 25418645

[B39] Powell F. L. Garcia N. (2000). Physiological effects of intermittent hypoxia. *High Alt. Med. Biol.* 1 125–136. 10.1089/15270290050074279 11256564

[B40] Rasmussen P. Siebenmann C. Diaz V. Lundby C. (2013). Red cell volume expansion at altitude: a meta-analysis and Monte Carlo simulation. *Med. Sci. Sports Exerc.* 45 1767–1772. 10.1249/MSS.0b013e31829047e5 23502972

[B41] Richalet J. P. Donoso M. V. Jimenez D. Antezana A. M. Hudson C. Cortes G. (2002). Chilean miners commuting from sea level to 4500 m: a prospective study. *High Alt. Med. Biol.* 3 159–166. 10.1089/15270290260131894 12162860

[B42] Sarybaev A. S. Palasiewicz G. Usupbaeva D. A. Plywaczewski R. Maripov A. M. Sydykov A. S. (2003). Effects of intermittent exposure to high altitude on pulmonary hemodynamics: a prospective study. *High Alt. Med. Biol.* 4 455–463. 10.1089/152702903322616209 14672548

[B43] Serebrovskaya T. V. Xi L. (2016). Intermittent hypoxia training as non-pharmacologic therapy for cardiovascular diseases: Practical analysis on methods and equipment. *Exp. Biol. Med.* 241 1708–1723. 10.1177/1535370216657614 27407098 PMC4999622

[B44] Siques P. Brito J. Leon-Velarde F. Barrios L. De La Cruz J. J. Lopez V. (2007). Hematological and lipid profile changes in sea-level natives after exposure to 3550-m altitude for 8 months. *High Alt. Med. Biol.* 8 286–295. 10.1089/ham.2007.8405 18081504

[B45] Skjelbakken T. Dahl I. M. S. Lochen M.-L. (2010). Changes in body mass index and smoking habits have a different impact on hemoglobin concentration in men and women: a longitudinal follow-up of the Tromso Study, 1994-2002. *Gender Med.* 7 230–239. 10.1016/j.genm.2010.06.006 20638628

[B46] Vinnikov D. Brimkulov N. Redding-Jones R. (2011). Four-year prospective study of lung function in workers in a high altitude (4000 m) mine. *High Alt. Med. Biol.* 12 65–69. 10.1089/ham.2010.1033 21452967

[B47] Wachsmuth N. B. Volzke C. Prommer N. Schmidt-Trucksass A. Frese F. Spahl O. (2013). The effects of classic altitude training on hemoglobin mass in swimmers. *Eur. J. Appl. Physiol.* 113 1199–1211. 10.1007/s00421-012-2536-0 23138148

[B48] West J. B. (2002). Intermittent exposure to high altitude. *High Alt. Med. Biol.* 3 141–143. 10.1089/15270290260131858 12162859

[B49] West J. B. (2004). The physiologic basis of high-altitude diseases. *Ann. Intern. Med.* 141 789–800. 10.7326/0003-4819-141-10-200411160-00010 15545679

[B50] West J. B. (2012). High-altitude medicine. *Am. J. Respir. Crit. Care Med.* 186 1229–1237. 10.1164/rccm.201207-1323CI 23103737

